# Carcinoid Tumor in a Mature Thymic Teratoma: A Rare Type of Extra Cardiac Mass

**DOI:** 10.1155/2012/507973

**Published:** 2012-10-08

**Authors:** Gregoriana Zanini, Mariella Chiudinelli, Luisa Bercich, Alessandra Pelati, Monica Bortolotti, Gian Franco Pasini

**Affiliations:** ^1^U.O.C. Cardiologia, Ospedale la Memoria di Gavardo, Via Gosa n. 74, 25085 Gavardo, Italy; ^2^Servizio di 1 Anatomia Patologica, Spedali Civili di Brescia (Brescia), Italy

## Abstract

We describe the case of a young female affected by a thymus teratoma coexisting with carcinoid tumor.

## 1. Introduction

 Malignant transformation of mature cystic teratoma is a rare complication occurring in approximately 1–3% of patients who have mature cystic teratoma. While any of the constituent tissues of a teratoma has the potential to undergo malignant transformation, squamous cell carcinoma is the most commonly associated malignancy [1]. The majority of mediastinal teratomas are mature teratomas that are histologically well defined and benign. Also mature teratomas do have the potential to undergo malignant transformation into a variety of malignancy (adenocarcinoma, rhabdomyosarcoma, leukemia) [1]. Thymic carcinoid is a malignant tumor: metastasis is common and the treatment is complete surgical resection. The prognosis of these tumors, when metastasis occur, are poor but difficult to assess because of little report in literature. Some authors described in kidneys a better prognosis for carcinoid tumor arising within teratomatous lesion compared to those arising within normal kidneys [2]. Thymus carcinoid tumors are rare and frequently associated with serotonin and neurotensin secretion. Thymus teratoma rarely presents together with carcinoid tumor. To the best of our knowledge, there has been only a report of thymus teratoma coexisting with carcinoid tumor [3].

## 2. Case 

**Figure 1:**
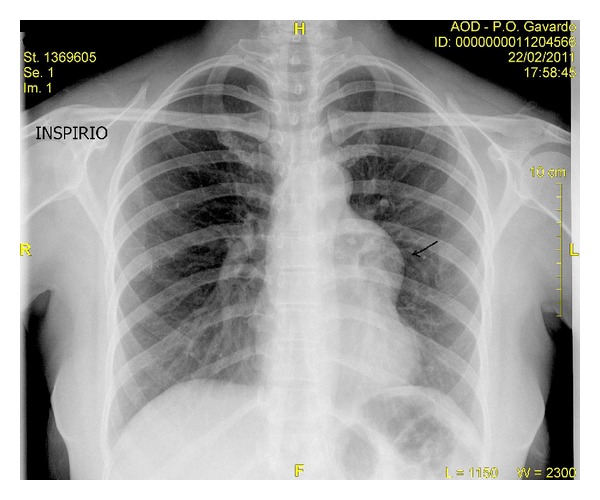
Chest X-ray in P-A: opacity mass of about 4.5 cm skull-caudal extension in left lateral anterior mediastinum.

**Figure 2:**
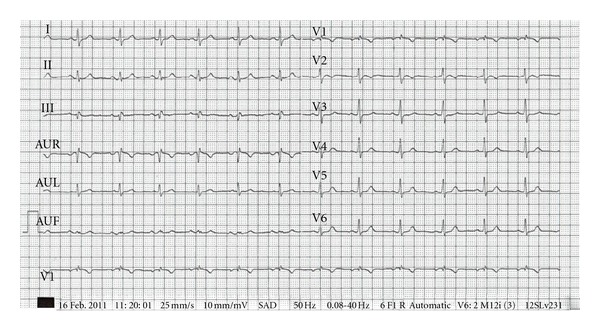
Electrocardiogram is normal.

**Figure 3:**
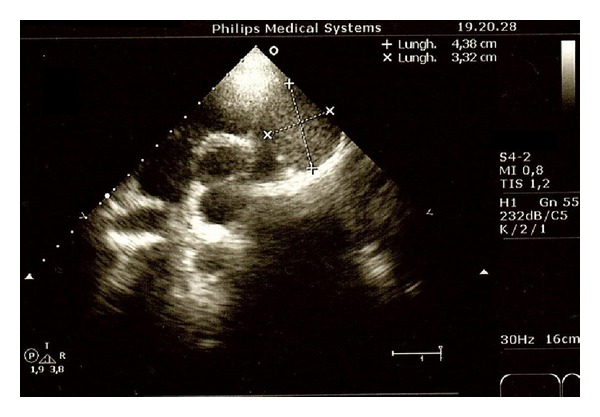
Transthoracic echocardiography with an ovalar iperecogenous mass of about 3.4 × 4.3 cm near the left side of the pulmonary trunk.

**Figure 4:**
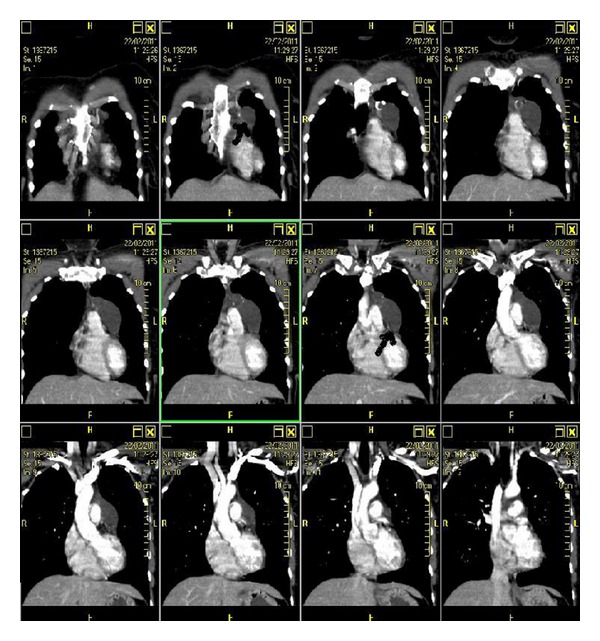
Thoracic medium contrast computerized axialtomography: encapsulated dishomogeneous expansive anterior mediastinal lesion of diameter 5.0 × 7.5 cm, with some calcification.

**Figure 5:**
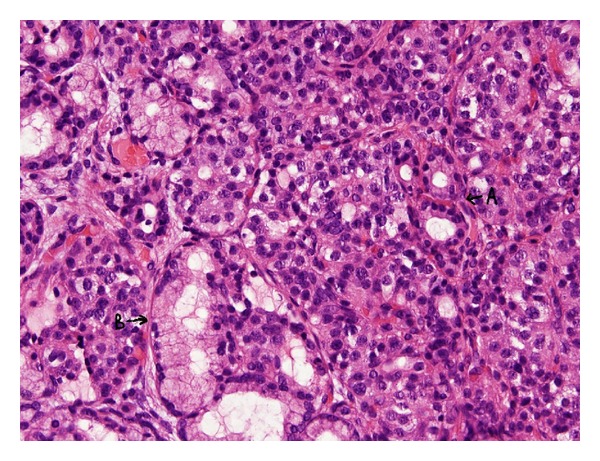
Microscopically (hematoxylin/eosin and cromogranina immunohistochemical staining): coexistent mature cystic teratoma (A) and moderately differentiated carcinoid tumor (B).

**Figure 6:**
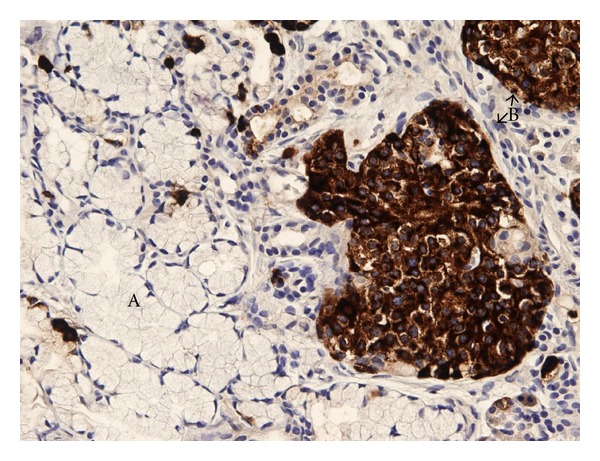
Microscopically (hematoxylin/eosin and cromogranina immunohistochemical staining): coexistent mature cystic teratoma (A) and moderately differentiated carcinoid tumor (B).

We present a rare case of typical carcinoid tumor arising within mature cystic teratoma of thymus in a 38-year-old female. Chest X-ray, done for incessancy cough, accidentally found an enlargement of the anterior mediastinum (Figure 1). Further ECG (Figure 2), transthoracic echocardiography, and then transoesophageal one (Figure 3) revealed an ovalar extracardiac mass immediately anterior to the first tract of the pulmonary artery and anterior to the left atrium without compression effect. She also underwent a thoracic medium contrast computerized axialtomography (Figure 4) that confirmed the encapsulated dishomogeneous expansive anterior mediastinal lesion of diameter 5.0 × 7.5 cm, with some calcification. She underwent to a surgical treatment with total thymectomy by left axillary thoracotomy saving muscles. Macroscopically the single chambered cystic lesion of about 8 × 6 × 3.7 cm was encapsulated and multiloculated with brown smooth thick wall filled with yellow-tan material. Inside the wall, there was a 1–1.5 cm nodular white area with calcification. Microscopically, the tumor showed coexistent mature cystic teratoma and moderately differentiated carcinoid tumor (Figures 5 and 6). The teratomatous cysts and carcinoid tumor showed strong staining for pankeratin, chromogranin and synaptophysin and insulin; negative for PTH, TTF1, gastrin, glucagon. 

## 3. Discussion and Conclusion

This very rare case of extra cardiac mass emphasizes the need for thorough sectioning and entire submission for histologic evaluation of all the mass in particular of mature cystic teratomas, in order to avoid missing multiple additional histogenetically distinct neoplasms. The treatment for early-stage carcinoid tumors is surgery alone and excellent outcomes can be expected in these cases [4].
